# Correlation of Radiographic and Palpable Landmarks Used for Tibial Plateau Levelling Osteotomy Planning

**DOI:** 10.1055/a-2789-2423

**Published:** 2026-03-31

**Authors:** Christian A. Folk, Ursula Krotscheck

**Affiliations:** Department of Small Animal Clinical Sciences, Cornell University College of Veterinary Medicine, Ithaca, New York, United States

**Keywords:** dog, stifle, cranial cruciate ligament, tibial plateau leveling osteotomy, radiographic planning

## Abstract

**Objective:**

To determine the correlation between preoperative and intraoperative tibial plateau leveling osteotomy (TPLO) planning landmarks and measurements.

**Study Design:**

In canine cadaveric stifles ( *n* = 39), the medial collateral ligament (MCL), palpable tibial tuberosity (pTT), patellar ligament enthesis (PLE), and the medial anatomic groove caudal to the attachment of the (patellar) ligament (MAGCAL) were labeled and radiographed. The location of the pTT and PLE were compared with the radiographic location of the tibial tuberosity (rTT); the MCL location was compared with the radiographic location of the intercondylar eminence (ICE), and the planned osteotomy location was compared with the MAGCAL position.

**Results:**

There was no statistically significant difference between the location of the rTT to the pTT and PLE. The MCL was directly centered over the ICE in only 20/39 stifles, with the rest being caudal. The planned osteotomy was cranial to or within the MAGCAL in 27/32 pelvic limbs.

**Conclusion:**

The MCL should be used cautiously for osteotomy planning, and the MAGCAL may be a useful anatomic landmark for intraoperative TPLO planning.

## Introduction

Cranial cruciate ligament insufficiency is the most common non-traumatic cause of hind limb lameness in dogs, with the tibial plateau leveling osteotomy (TPLO) being one of the primary surgical treatment options for affected patients. 1 2 3 4 This periarticular procedure stabilizes the stifle joint during weight bearing without replacing the ligament, but instead by reducing the tibial plateau angle (TPA) through a proximal tibial osteotomy and predetermined rotation of the cut segment. Reduction of the TPA ultimately eliminates cranial tibial subluxation (thrust) during axial loading, reducing the progression of osteoarthritis in canine patients with cranial cruciate ligament insufficiency. When performing the tibial osteotomy, centering the osteotomy on the proximal end of the tibial long axis (TLA), radiographically defined by a point overlying the intercondylar eminence (ICE), prevents translation of the TLA following rotation of the proximal segment, resulting in a final TPA similar to the target degree. 5 6

Pre- and intraoperative planning has been shown to increase the likelihood of achieving a centered osteotomy during a TPLO. 7 8 9 This can be accomplished using previously published methods that utilize radiographic and intraoperative anatomic landmarks on the canine stifle, with or without the aid of an alignment jig, cutting guide, Kirschner wires, or some combination of these. 6 7 8 9 10 11 12 13 The commonly used anatomic landmarks include the tibial attachment of the patellar ligament, best appreciated by the patellar ligament enthesis (PLE), which is believed to correlate with the prominent, radiographic tibial tuberosity (rTT) and the medial collateral ligament (MCL) which has been assumed to overly the intercondylar eminence (ICE). 7 9 10 11 13 Previous studies have recommended centering the TPLO cut at the ICE, a reliable radiographic landmark that roughly estimates the center of rotation of the stifle joint in patients with cranial cruciate ligament insufficiency. 5 11 12 13 14 However, the accuracy and correlation of these individual radiographic and intraoperative landmarks used for TPLO planning have not been assessed. The accuracy of an added landmark that is used for intraoperative determination of TPLO cut location has not been evaluated, namely, the depression at the cranial–proximal aspect of the tibia, where the tibial tuberosity meets the tibial plateau or the “medial anatomic groove caudal to the attachment of the (patellar) ligament (MAGCAL).”

The purpose of this study was (1) to determine the correlation between radiographic and intraoperative surgical TPLO planning landmarks and (2) to determine if the MAGCAL is a valuable landmark in TPLO planning. We hypothesized that the palpable tibial tuberosity (pTT), PLE, MCL, and MAGCAL would show anatomic variability between canine stifles and thus would not be accurate identifiers of the rTT, ICE, and osteotomy position, respectively.

## Methods and Materials

### Specimen Preparation

Routine craniomedial approaches to the proximal tibiae were performed using 39 cadaveric canine pelvic limbs, allowing for exposure of the MCL, PLE, and the cranial-most aspect of the tibial tuberosity. 2 6 11 12 The joint capsule remained intact while labeling the anatomic landmarks. The MCL was identified, and its entire width was sharply scored along its long axis with a #15 blade and coated with barium sulfate cream (E-Z-Paste, barium sulfate esophageal cream [60% w/w], E-Z-EM Canada Inc., Montreal, QC, Canada) for identification on preoperative planning radiographs. The prominent, palpable tibial tuberosity (pTT) was found by exposure and palpation of the cranial-most aspect of the proximal tibia. The patellar ligament entheses (PLE) were exposed, appearing as a white line crossing the tibial tuberosity. Both landmarks were marked with 21- and 18-gauge hypodermic needles for radiographic identification. Only a single hypodermic needle was placed if both PLE and pTT were appreciated to be in the exact same location. An additional landmark labeled for the current study is the palpable depression present along the craniomedial aspect of the proximal tibia, where the tibial tuberosity joins the joint surface of the tibial plateau. To the authors' knowledge, there is currently no anatomic description for this landmark; therefore, it was labeled as the medial anatomic groove caudal to the attachment of the (patellar) ligament (MAGCAL). 15 After identification, its palpable width was sharply scored with a #15 blade and marked using barium sulfate cream, highlighting the depressed region of the bone. Any excess barium sulfate cream that was not within the visible or palpable depression was removed from the surface of the tibiae. Once all anatomic landmarks were labeled with radiopaque medium, calibrated digital radiographic projections (lateral flexed 90–90 stifle and tarsus and craniocaudal views) of the stifles were obtained as previously described and imported into a commercially available digital templating software (vPOP, VETSOS Education Ltd, England and Wales, UK) for preoperative TPLO planning. 2 5 6 7 8 9 10 11 13 In an attempt to standardize landmark measurements based on the dog's size, the length of the TLA (in millimeters) was acquired in all tibiae used, which was defined as a line drawn from the ICE to the center of the talus, with the actual measurement ending at the distal-most aspect of the tibiae. The distances from the gross, palpable surgical landmarks to their “equivalent” radiographic locations were measured. Specifically, distances from the PLE and pTT to the rTT, and from the center of the MCL to the ICE were measured. Negative numbers were used to indicate whether the gross surgical landmark (pTT and PLE) was distal to the rTT or if the MCL was caudal to the ICE. A TPLO was planned centered over the ICE, and the distance between the proximal tibial osteotomy exit and the cranial aspect of the MAGCAL was measured. Negative numbers denote if the planned osteotomy would be cranial to the cranial border of the MAGCAL. Using the previously described “D1, D2, and D3” measurements for preoperative and intraoperative osteotomy planning, D2 (rTT to proximal exit of the osteotomy) was compared with the MAGCAL location relative to the rTT (rTT to cranial aspect of MAGCAL). 6 8 9 10 For clarity, the D1 and D3 measurements were defined as the distance from the rTT to the most craniodistal aspect of the osteotomy and the distance from the MCL to the caudal aspect of the osteotomy, respectively.

### Statistical Analysis

Statistical analysis was performed using commercial statistical software (IBM SPSS Statistics 26.0 for Windows, IBM, Armonk, NY, USA). The Shapiro-Wilk test was used to assess data normality. Parametric data were presented as a mean ± SD, and non-parametric data were expressed as median (range). Categorical data were expressed as frequencies and percentages. Continuous data were compared using a one-sample *t* -test and Wilcoxon signed rank test for normal and non-normally distributed data, respectively. Effect sizes were calculated using Cohen's d and r for parametric and non-parametric data, respectively, with small, medium, and large effects corresponding to d values of 0.2, 0.5, and 0.8 and r values of 0.1, 0.3, and 0.5. 16 Statistical significance was identified at a *P* -value of <0.05.

## Results

A total of 39 pelvic limbs were included in the study (20 left, 19 right). The median length of the TLA was 180 mm (165–240 mm; *p* ≤ 0.001). Two of the pelvic limbs showed radiographic signs of cranial cruciate ligament disease, including cranial tibial thrust and stifle osteoarthrosis, which was confirmed via medial arthrotomies. Inspection of the stifle joints revealed intact cranial cruciate ligaments in all other pelvic limbs. In seven limbs, the contrast on the MAGCAL did not define it appropriately. Therefore, these were excluded from any analysis assessing the MAGCAL.

### Medial Collateral Ligament versus Intercondylar Eminence

**Fig. 1:**
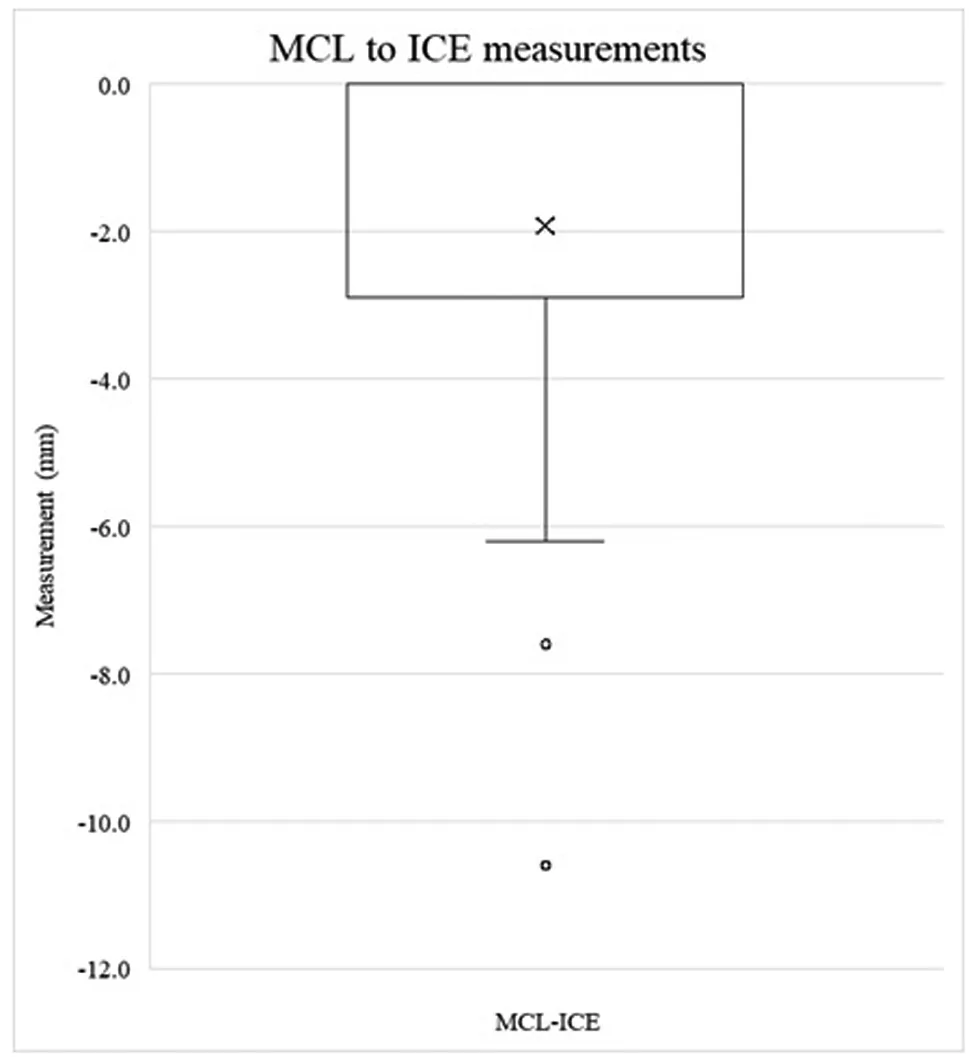
Box and whisker plot demonstrating the distribution of measurements between the medial collateral ligament (MCL) and the intercondylar eminence (ICE). Negative values indicate that the MCL is caudal to the ICE. The box represents the interquartile range with medians marked by a horizontal line within the box. The whisker indicates the range excluding the outliers, which are shown as individual points. The “x” within the box delineates the mean value. The outliers demonstrated here reflect the stifles with radiographic evidence of cranial cruciate ligament insufficiency and subsequently the most significant deviation of the MCL to the ICE.

**Fig. 2:**
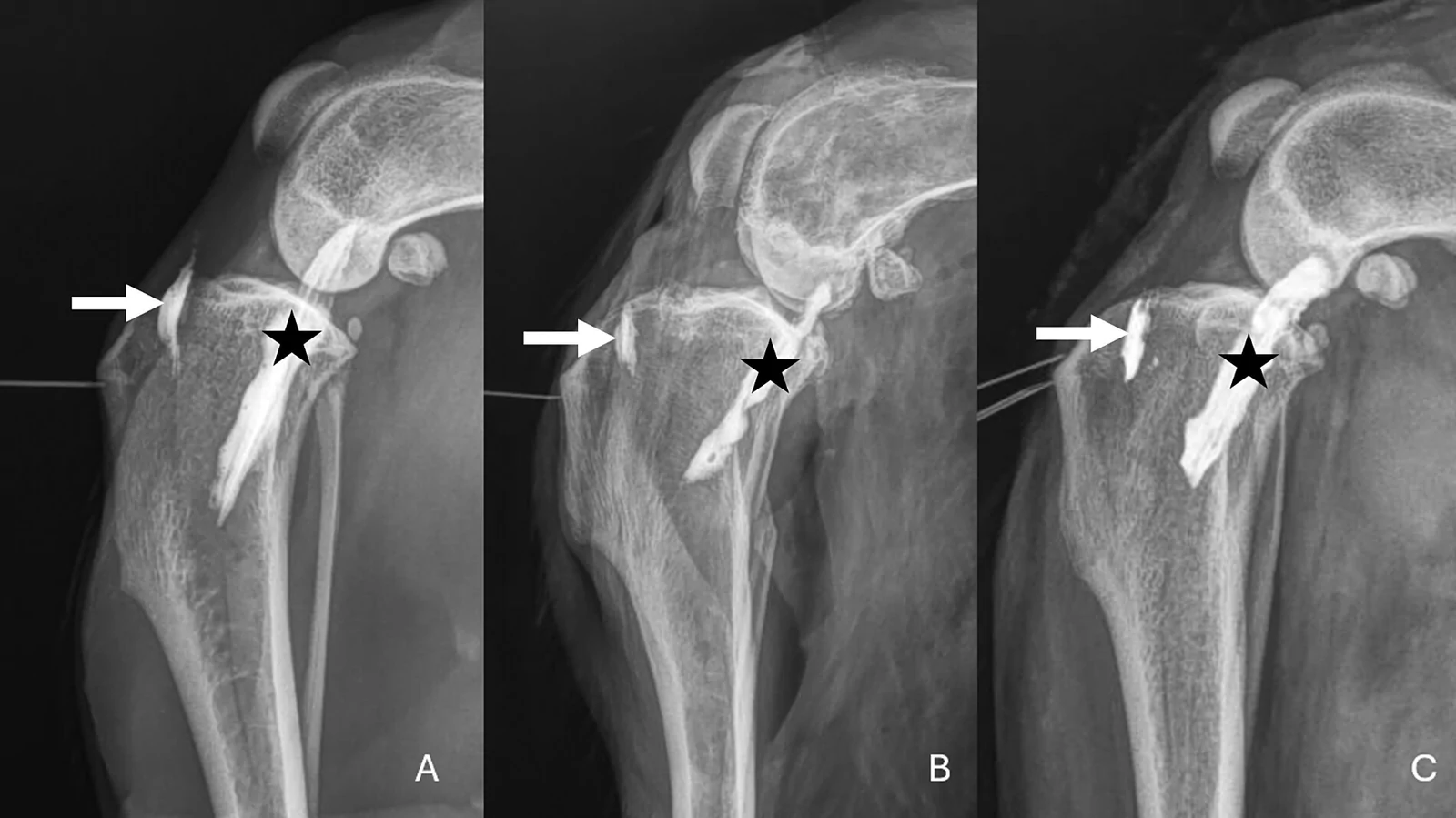
Lateral radiograph of the stifle with the medial collateral ligament (black star) spanning the joint space directly over the intercondylar eminence ( **A** ), caudal to the intercondylar eminence in a dog with radiographic evidence of cruciate disease ( **B** ), and caudal to the intercondylar eminence in a dog *without* radiographic evidence of cruciate disease ( **C** ). The medial anatomic groove caudal to the attachment of the (patellar) ligament (MAGCAL) (white arrow) has been scored and marked with barium sulfate cream.

In assessing the location of the MCL as it crosses the stifle joint in relation to the ICE, the MCL was directly centered over the ICE in 20/39 stifles of cadaveric canine stifles; the remainder were 2.9 mm (1.4–10.6 mm) caudal to the ICE ( Fig. 1 ). A Wilcoxon signed-rank test and effect size (r) were calculated to assess the significance of the median value from zero (the MCL being centered over the ICE). The test revealed a significant (caudal) deviation of the MCL from the ICE ( *p* ≤ 0.001) with a medium effect size (r = 0.68). The two pelvic limbs with radiographic evidence of cruciate disease exhibited MCL with the highest caudal deviation relative to the ICE with values of 7.6 and 10.6 mm ( Figs. 1 and 2 ). An equivalence test was performed to assess the relationship between the TLA length and the location of the MCL and ICE, revealing no significant relationship.

### Radiographic Tibial Tuberosity versus Palpable Tibial Tuberosity, Patellar Ligament Enthesis

**Fig. 3:**
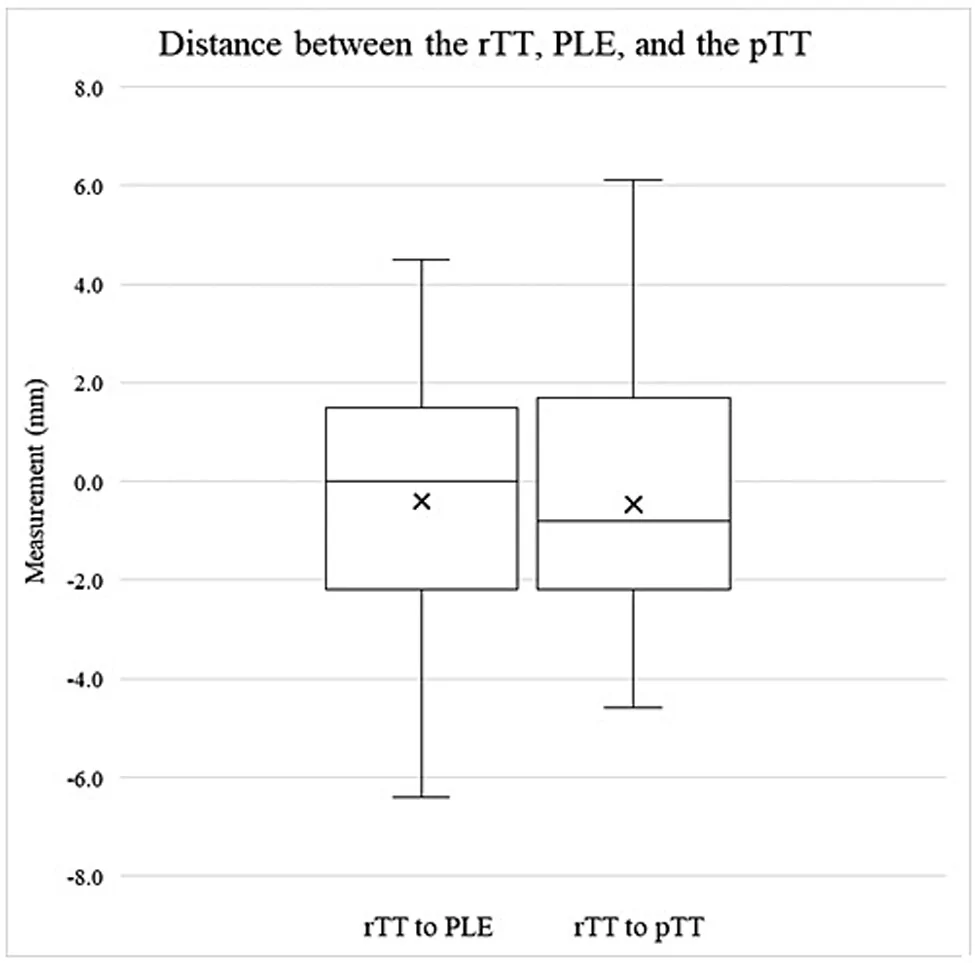
Box and Whisker plot demonstrating the radiographic tibial tuberosities' (rTT) location relative to the position of the patellar ligament enthesis (PLE) and the palpable tibial tuberosity (pTT). Negative values indicate a distal position, and positive values indicate a proximal position. The boxes represent the interquartile range with medians marked by a horizontal line within the boxes. The whiskers indicate the range. The “x” within the boxes delineates the mean values.

The rTT was typically distal to both PLE (0.4 ± 2.5 mm) and the pTT (0.5 ± 2.7 mm) ( Fig. 3 ). Only 10/39 and 7/39 rTT accurately represented the PLE and pTT, respectively; however, one-sample *t* -tests were conducted to determine whether the mean distances differed significantly from zero, with a value of zero indicating that the two landmarks are indistinguishable from one another. Neither comparison revealed a statistically significant difference (rTT to PLE: *p* = 0.32, Cohen's d = −0.16; rTT to pTT: *p* = 0.29; Cohen's d = −0.17). In 15/39 stifles, the pTT and PLE were identical.

### MAGCAL versus Planned Osteotomy

**Fig. 4:**
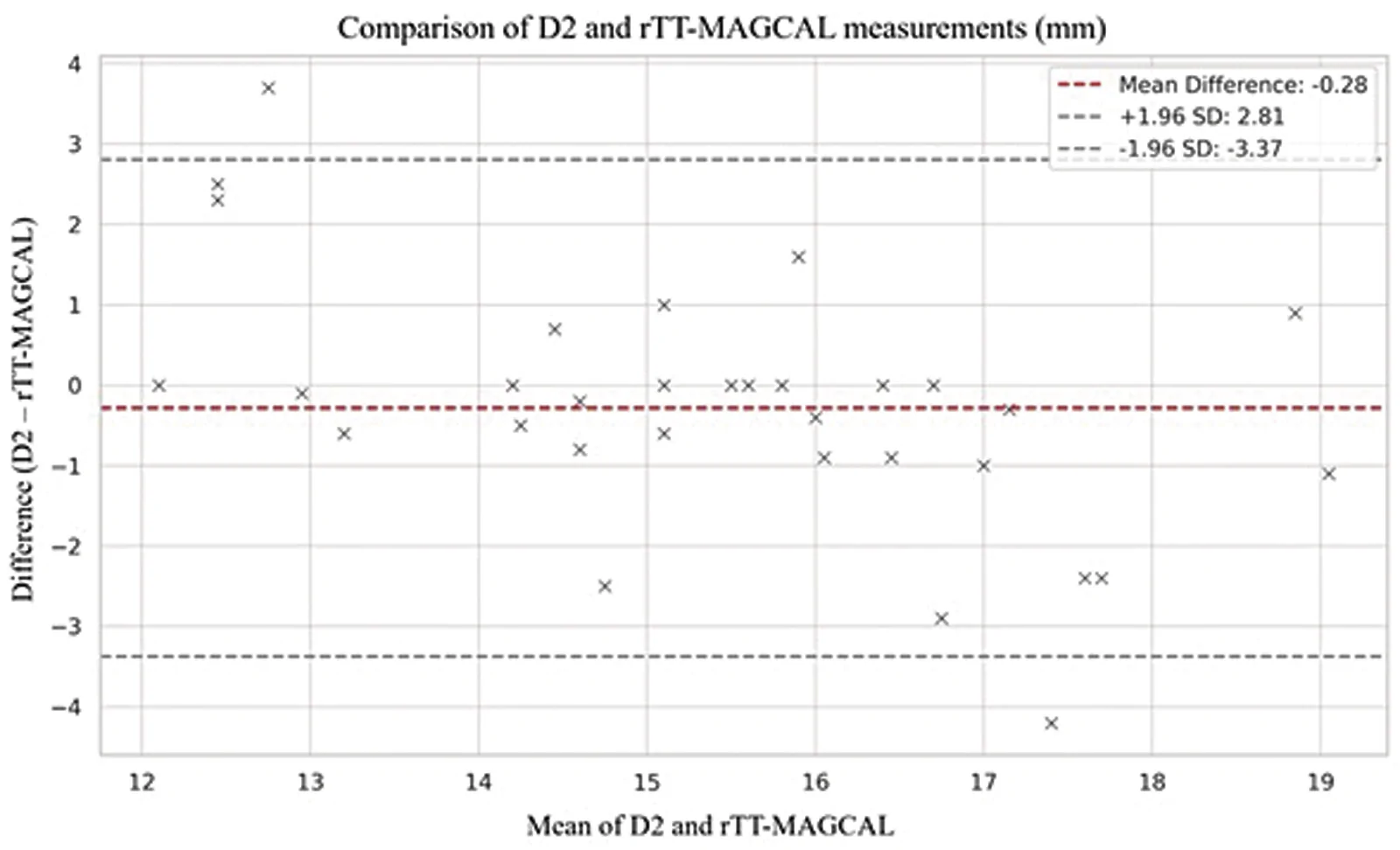
Bland-Altman chart illustrating the mean and difference between the D2 and rTT-MAGCAL measurements. There was no significant difference ( *p* = 0.315) between the two measurements, further illustrated by the low mean difference of 0.28 mm with 95% limits of agreement of 2.81 mm and −3.37 mm. MAGCAL, medial anatomic groove caudal to the attachment of the (patellar) ligament; rTT, radiographic tibial tuberosity.

Contrast was not clear at the level of the tibial plateau in seven specimens, which were excluded from this analysis ( *n* = 32). Mean (SD) for D2 was 15.4 (± 1.7) mm and the distance between the rTT and the MAGCAL was 15.6 (± 2.3) mm. Paired-samples *t* -test of D2 and the distance from the rTT to the cranial aspect of the MAGCAL (rTT-MAGCAL) showed no significant differences between the two measurements ( *p* = 0.32). The agreement between D2 and rTT-MAGCAL was assessed using a Bland-Altman plot ( Fig. 4 ). Proportional bias was assessed by performing a linear regression of the difference (D2 − rTT-MAGCAL) against the mean of the two measurements (D2 and rTT-MAGCAL), which demonstrated a significant slope (β = −0.45, 95% CI −0.18 to −0.73, *p* = 0.002). These findings confirmed a proportional bias between the D2 and rTT-MAGCAL measurements with smaller means tending to yield positive differences and larger means producing more negative differences. When assessing the planned osteotomy relative to the MAGCAL, 26/32 of planned osteotomies were within 2 mm of the cranial aspect of the MAGCAL. Of the remaining six osteotomies, three were more than 2 mm cranial to the cranial aspect of the MAGCAL, and three were more than 2 mm caudal to the cranial aspect but still within the MAGCAL. None of the osteotomies were caudal to the MAGCAL.

Following the initial statistical analyses, an assessment was made to determine whether the TLA length (and indirectly, the size of the canine patient) would affect the distances between the assessed anatomical landmarks. Linear regression models were performed with TLA as the predictor variable and each landmark distance as the dependent variable. The TLA length was not significantly associated with the MCL-ICE ( *p* = 0.85) or rTT-SF ( *p* = 0.20) measurement; however, it was associated with the rTT-pTT measurement ( *p* = 0.012).

## Discussion

The present study describes the anatomic variability of specific radiographic and intraoperative landmarks of the canine stifle. Given the results, the hypothesis was partially accepted, as the MCL was not a consistently accurate identifier of the ICE. The patellar ligament enthesis and the pTT were more reliable predictors of the rTT; however, minor, non-significant variations exist depending on the patient's size, with the pTT and PLE being commonly located proximal to the rTT. The use of the pTT and/or PLE as intraoperative landmarks to create the D1 and D2 measurements could theoretically result in a more proximal osteotomy, leading to caudoproximal movement of the proximal tibial long axis point and subsequent over-rotation, as well as a decrease in the postoperative TPA. 5 Depending on the degree of rotation and size of the patient, this inaccuracy could lead to excessive strain on the caudal cruciate ligament and an osteotomy that leads to less available bone for the placement of a TPLO bone plate; however, a mean difference of 0.5 mm for both locations may not be clinically relevant and cause a negligible difference in the executed rotation and subsequent TPA. When accounting for tibial size using the TLA length, a significant correlation was observed between the distance of the rTT and pTT, suggesting that more caution should be exercised when using these landmarks synonymously in patients of both smaller and larger sizes.

The MCL is an unreliable intraoperative anatomic landmark, with only half appropriately delineating the position of the ICE. Using the MCL as a translational anatomic reference for the ICE to portray the D3 measurement intraoperatively could lead to a caudal and/or distal osteotomy in 50% of stifles. Placement of the osteotomy such that it is centered distal or caudal to the proximal tibial long axis point could result in an increase or decrease in the postoperative TPA, respectively, following rotation. Interestingly, a previous study discussing the effects of osteotomy location on the rotation of the proximal tibial segment describes unpublished data showing a distal osteotomy as the most common location in their clinical patients. 5 Our results indicate that cadavers with radiographic evidence of cruciate disease exhibit the highest degree of caudal MCL deviation, which could be a potential explanation for distal osteotomies. A cadaveric study assessing different locations for reconstruction of the medial collateral ligament revealed cranial positioning of the MCL to the intercondylar eminence with the stifle in terminal extension and caudal positioning in maximal flexion. 17 However, that study did not assess the location of the MCL with the stifle in 90 degrees of flexion, unlike the limbs used in the current study. The use of the MCL as an intraoperative landmark for the ICE, particularly in patients with significant tibial thrust, may be an inaccurate intraoperative identifier of the ICE for osteotomy planning. These findings appear to be independent of the patient's size based on the lack of correlation to their TLA lengths in the current study.

The present study suggests that the MAGCAL may serve as an additional anatomic landmark for surgeons to use intraoperatively when planning and verifying their osteotomy measurements, specifically the D2 measurement. In the limbs evaluated, the planned osteotomy was consistently cranial to or within the MAGCAL. In these cases, placement of the osteotomy too far cranial could result in a tibial tuberosity with little width, leading to postoperative complications such as a tibial tuberosity fracture. 18 However, based on the data presented, it is recommended that osteotomies remain within or slightly cranial to the MAGCAL, not caudal. An additional word of caution when using the MAGCAL as an identifiable landmark for D2 would be the awareness that as the values get smaller or larger (secondary to the size of the limb), the difference between the two measurements (rTT-MAGCAL and D2) begins to widen significantly; however, the impact of these few millimeters in a clinical setting is not known and likely to vary depending on the size of the patient.

There are multiple limitations associated with the study, including the use of primarily normal canine cadavers. The weight, sex, and breed of the cadaveric limbs were unknown at the time of data collection; however, an effort was made to standardize the landmark measurements using the TLA length while acknowledging the variability in the size of the tibiae used. A difference in assessment of anatomic variability may also exist when assessing only stifles with cruciate disease as preoperative radiographic and intraoperative landmarks could be more challenging to determine with the presence of osteoarthritis and thickening and/or fibrosis of the joint capsule. Using cadaveric limbs may have also provided stifle joints with increased laxity due to natural tissue degradation after freezing and thawing the specimens for use. These factors could have contributed to the malpositioning of the femurs, tibiae, or MCL, resulting in the data presented. Other limitations include a single observer obtaining all preoperative and postoperative planning measurements to limit any variability; hence, further studies to evaluate intra- and interobserver variability may be warranted. The observer obtaining the preoperative and postoperative measurements was not the same person performing the intraoperative labeling of anatomic landmarks or the osteotomies.

## Conclusion

In conclusion, the relationship between PLE, pTT, and rTT is somewhat inconsistent and should be used with caution for osteotomy planning, especially in smaller or larger breed canine patients. The use of the medial collateral ligament to identify the center of rotation is only accurate in 50% of cadaver limbs. The number of cadaver limbs with pre-existing osteoarthritis and cranial cruciate ligament disease in this study is limited; however, our findings suggest caudal displacement of the MCL to be more pronounced in that subset of patients. Lastly, the MAGCAL may be an additional useful anatomic landmark that ensures the planned osteotomy is cranial to or within the notch, thus avoiding the joint surface.

## References

[1] Witsberger T H Villamil J A Schultz L G Hahn A W Cook J L Prevalence of and risk factors for hip dysplasia and cranial cruciate ligament deficiency in dogs J Am Vet Med Assoc 2008 232 12 1818 1824 18598150 10.2460/javma.232.12.1818

[2] Slocum B Slocum T D Tibial plateau leveling osteotomy for repair of cranial cruciate ligament rupture in the canine Vet Clin North Am Small Anim Pract 1993 23 04 777 795 8337790 10.1016/s0195-5616(93)50082-7

[3] Gatineau M Dupuis J Planté J Moreau M Retrospective study of 476 tibial plateau leveling osteotomy procedures Vet Comp Orthop Traumatol 2011 24 333 341 21792470 10.3415/VCOT-10-07-0109

[4] Johnson J A Austin C Breur G J Incidence of canine appendicular musculoskeletal disorders in 16 veterinary teaching hospitals from 1980 through 1989 Vet Comp Orthop Traumatol 1994 7(2) 56 69

[5] Kowaleski M P McCarthy R J Geometric analysis evaluating the effect of tibial plateau leveling osteotomy position on postoperative tibial plateau slope Vet Comp Orthop Traumatol 2004 17(1) 30 34

[6] Kowaleski M P Stifle joint St. Louis Saunders 2018 1115 1120

[7] Collins J E Degner D A Hauptman J G DeCamp C E Benefits of pre- and intraoperative planning for tibial plateau leveling osteotomy Vet Surg 2014 43 02 142 149 24491234 10.1111/j.1532-950X.2013.12093.x

[8] Mossman H von Pfeil D JF Nicholson M Accuracy of three pre- and intra-operative measurement techniques for osteotomy positioning in the tibial plateau levelling procedure Vet Comp Orthop Traumatol 2015 28 04 250 255 26167862 10.3415/VCOT-14-12-0188

[9] Woodbridge N Knuchel-Takano A Brissot H Nelissen P Bush M Owen M Accuracy evaluation of a two-wire technique for osteotomy positioning in the tibial plateau levelling procedure Vet Comp Orthop Traumatol 2014 27 01 8 13 24146104 10.3415/VCOT-13-01-0014

[10] Talaat M B Kowaleski M P Boudrieau R J Combination tibial plateau leveling osteotomy and cranial closing wedge osteotomy of the tibia for the treatment of cranial cruciate ligament-deficient stifles with excessive tibial plateau angle Vet Surg 2006 35 08 729 739 17187635 10.1111/j.1532-950X.2006.00217.x

[11] Tan C J Bergh M S Schembri M A Johnson K A Accuracy of tibial osteotomy placement using 2 different tibial plateau leveling osteotomy jigs Vet Surg 2014 43 05 525 533 24617418 10.1111/j.1532-950X.2014.12173.x

[12] Craig A Witte P G Scott H W Sagittal accuracy of tibial osteotomy position during in vivo tibial plateau levelling osteotomy performed without an alignment jig and cutting guide Vet Comp Orthop Traumatol 2017 30 01 37 45 27849109 10.3415/VCOT-16-01-0002

[13] Grierson J Sanders M Guitan J Pead M Comparison of anatomical tibial plateau angle versus observer measurement from lateral radiographs in dogs Vet Comp Orthop Traumatol 2005 18 04 215 219 16594389

[14] Kowaleski M P Apelt D Mattoon J S Litsky A S The effect of tibial plateau leveling osteotomy position on cranial tibial subluxation: an in vitro study Vet Surg 2005 34 04 332 336 16212587 10.1111/j.1532-950X.2005.00051.x

[15] Hermanson J W De Lahunta A Evans H E Millers and Evans' Anatomy of the Dog 5th ed. St. Louis Elsevier 2020 164 167

[16] Cohen J Statistical Power Analysis for the Behavioral Sciences 2nd ed. Hillsdale L. Erlbaum Associates 1988 531 534

[17] Fischer C Alarcon J C Troncoso I E Cherres M D An in vitro study of potential attachment sites for the reconstruction of the medial collateral ligament of canine stifles Arch Med Vet 2015 47 259 262

[18] Bergh M S Rajala-Schultz P Johnson K A Risk factors for tibial tuberosity fracture after tibial plateau leveling osteotomy in dogs Vet Surg 2008 37 04 374 382 18564262 10.1111/j.1532-950X.2008.00391.x

